# Benchmarking Multilayer Perceptron Configurations for Damage Classification in UAV Composite Wings Using Fiber Bragg Gratings Sensors

**DOI:** 10.3390/s26113377

**Published:** 2026-05-26

**Authors:** David O. Briceño González, Julian Sierra-Perez, Maribel Anaya Vejar, Diego Tibaduiza Burgos

**Affiliations:** 1 Departamento de Ingeniería Eléctrica y Electrónica, Universidad Nacional de Colombia, Bogotá 111321, Colombia; dbriceno@unal.edu.co (D.O.B.G.); manaya@unal.edu.co (M.A.V.); 2 Department of Aerospace Materials and Processes, Universidad Politécnica de Madrid, 28040 Madrid, Spain; juliansierrap@gmail.com

**Keywords:** Fiber Bragg Grating (FBG) sensors, UAV composite wings, structural health monitoring (SHM), multilayer perceptron (MLP), benchmarking, damage classification

## Abstract

**Highlights:**

**What are the main findings?**

- A systematic benchmarking of Multilayer Perceptron (MLP) configurations was conducted using real strain data from a composite UAV wing instrumented with 32 Fiber Bragg Grating (FBG) sensors.
- Adaptive optimizers (Nadam and AdamW) and compact architectures (256–128–64) achieved the best trade-off between accuracy, stability, and computational efficiency.

**What are the implications of the main findings?**

- Results demonstrate that lightweight neural architectures can reliably classify structural damage under sensor uncertainty, enabling practical implementation in embedded SHM systems for UAVs.
- The proposed benchmarking framework provides reproducible guidelines for model selection in data-driven SHM, supporting future integration with digital twin and real-time diagnostic applications.

**Abstract:**

Structural damage classification in composite UAV wings is a key challenge in Structural Health Monitoring (SHM), particularly under barely visible impact damage conditions. Fiber Bragg Grating (FBG) sensor networks provide high-resolution strain data; however, systematic experimental benchmarking of lightweight neural architectures trained on real FBG datasets remains limited, especially under sensor degradation scenarios. This work presents a four-phase benchmarking study of Multilayer Perceptron (MLP) configurations using strain measurements from a composite UAV wing instrumented with 32 FBG sensors across five damage states and 210 loading experiments. The framework evaluates optimization strategies, hyperparameter sensitivity, architectural depth, and robustness under controlled sensor dropout, Gaussian noise, and wavelength drift perturbations. Results indicate that compact architectures with progressive dimensional reduction (256–128–64) trained using adaptive optimizers (AdamW and Nadam) achieve the best balance between macro-F1 performance (up to 0.85 during validation), stability, and computational efficiency. Robustness analysis shows gradual performance degradation under sensor loss, suggesting distributed strain-field learning. These findings provide practical guidelines for selecting computationally efficient and robust neural models for deployable FBG-based SHM systems in aerospace applications.

## 1. Introduction

Low-velocity impacts of composite structures produce barely visible impact damage (BVID), which may significantly compromise structural integrity and flight safety [1]. Because such damage can remain hidden within internal layers, reliable detection and localization techniques are essential [2]. While classical nondestructive testing (NDT) methods have traditionally been employed, modern Structural Health Monitoring (SHM) systems integrate sensors, data acquisition, and intelligent algorithms to enable continuous, real-time structural assessment [2].

### 1.1. Evolution of SHM and Data Driven Methods

The evolution of SHM technologies has involved the integration of advanced sensing strategies with data-driven analysis methods. Various sensor types have been employed in SHM applications [3]. Among them, Fiber Bragg Grating (FBG) sensors have become a key enabler due to their high sensitivity, multiplexing capability, and immunity to electromagnetic interference [4]. These characteristics make FBGs particularly suitable for aerospace applications, where lightweight and distributed sensing solutions are required [4,5]. Their use has been reported in spacecraft and adaptive composite structures for strain and temperature monitoring under harsh operational conditions [6].

The increasing availability of dense sensor networks has encouraged the adoption of machine learning (ML) techniques to extract meaningful information from high-dimensional strain data [7,8]. As noted in [9], SHM requires extracting damage-sensitive features from time-series measurements to determine structural states. Artificial Neural Networks (ANNs) and other surrogate modeling approaches have been explored to reduce computational cost while maintaining predictive capability, particularly in civil and aerospace structures [10].

Recent research has increasingly focused on hybrid and deep learning architectures for complex SHM scenarios. Examples include interpretable Lamb wave convolutional sparse coding methods for dispersive signals [11], hybrid CNN–LSTM frameworks for automatic feature extraction from multisensor time-series data [12], and metaheuristic-enhanced ANN approaches combining Rough Set methods and Support Vector Machines [13]. Optimization strategies such as hybrid swarm algorithms integrating PSO and BOA have also been proposed to enhance ANN training performance [14].

In addition, Bayesian Neural Networks have been explored for multi-class impact classification and uncertainty quantification [15]. Comparative studies evaluating ANN, CNN, and LSTM architectures under noise and uncertainty conditions have highlighted the importance of model selection in SHM tasks [16]. Embedded SHM systems using neural architectures have also demonstrated promising accuracy in composite joints [17]. Furthermore, CNN-based approaches trained on digital twin-generated data have shown strong performance for aircraft wing damage detection [18].

### 1.2. Research Gap and Motivation

Despite the extensive application of deep and hybrid architectures in SHM, several limitations remain. Many existing works rely on simulated datasets or digital twin environments [18], prioritize complex deep architectures with high computational cost [12], or focus primarily on accuracy improvements without systematic robustness evaluation under sensor degradation conditions [16].

There is still a limited number of systematic experimental benchmarking studies focused on lightweight Multilayer Perceptron (MLP) configurations trained on real FBG datasets under realistic scenarios of sensor dropout, noise, and wavelength drift. As a result, practical guidelines for selecting computationally efficient architectures suitable for embedded UAV SHM implementations remain insufficiently explored.

### 1.3. Previous Works and Contribution of This Study

Previous work by Sierra-Pérez et al. demonstrated the integration of FBG networks into composite UAV structures for real-time monitoring and damage detection [19]. Building upon this foundation, a supervised ANN model was later proposed to classify five damage states in a composite UAV wing instrumented with 32 FBG sensors, achieving strong classification performance on experimental data [20].

The present study extends these works by conducting a systematic benchmarking of Multilayer Perceptron (MLP) configurations using the same experimental dataset. The benchmarking protocol evaluates multiple optimizers, learning rates, architectural depths, and robustness under controlled perturbations.

The main contributions of this work are

- A four-phase reproducible benchmarking framework for evaluating MLP configurations in FBG-based SHM tasks.
- An experimental comparison of adaptive optimizers (Adam, Nadam, AdamW, RMSprop, Lion) using real composite wing data.
- A structured analysis of architectural complexity versus generalization performance.
- An explicit robustness evaluation under sensor dropout, Gaussian noise, and linear wavelength drift scenarios representative of operational uncertainty.

### 1.4. Structure of the Document

This work is organized as follows: first, a brief theoretical background is included in Section 2. Materials and methods are described in Section 3, where FBG-based SHM is briefly described, and the data preprocessing and damage classification pipeline are included. Also, the benchmarking methodology and the four progressive experimental phases are included. This section describes in the same way the specimen and materials used for the validation. Results are then included in Section 4 for each of the phases, and discussion and concluding remarks are finally given in the last section (Section 5).

## 2. Theoretical Background

### 2.1. FBG-Based SHM

**Figure 1:**
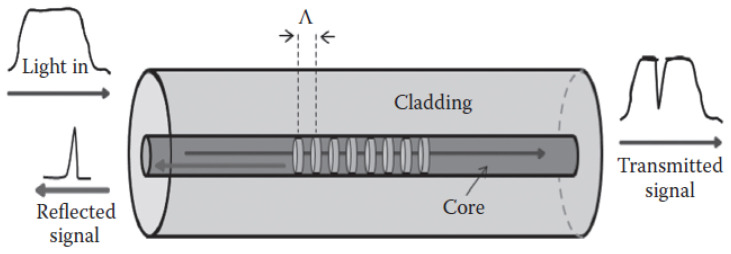
Schematic representation of a Fiber Bragg Grating (FBG) sensor showing the periodic modulation of the refractive index along the fiber core and the reflected Bragg wavelength $\lambda_{B}=2n_{\mathrm{eff}}\Lambda$ [2].

Fiber Bragg Grating (FBG) sensors are widely used in Structural Health Monitoring (SHM) systems for simultaneous strain and temperature measurement. Figure 1 shows a schematic representation of a Fiber Bragg Grating (FBG) sensor showing the periodic modulation of the refractive index along the fiber core and the reflected Bragg wavelength, this is explained as follows.

An FBG reflects a specific wavelength

(1) $$\lambda_{B}=2n_{\mathrm{eff}}\Lambda$$

that shifts linearly with variations in strain (ε) and temperature (ΔT), according to

(2) $$\frac{\Delta \lambda_{B}}{\lambda_{B}}=\left(1-p_{e}\right)\;\varepsilon +\left(\alpha +\xi \right)\;\Delta T$$

where $p_{e}$ is the photoelastic coefficient, α the thermal expansion, and ξ the thermo optic coefficient [5]. Thanks to their wavelength encoded response, FBG sensors offer high resolution, immunity to electromagnetic interference and multiplexing capability, enabling distributed sensing through wavelength division multiplexing (WDM) [4]. These features make them ideal for lightweight composite structures in aerospace applications such as wings, fuselage sections, and rotor blades [21]. FBGs have also been successfully deployed in spacecraft to monitor strain and temperature in critical components under harsh environments [22]; their integration into composite laminates allows continuous tracking of strain fields and early damage detection [21], supporting the development of data-driven SHM systems that combine optical sensing with machine learning-based diagnosis [7].

### 2.2. Optimizers

Optimizers in deep neural networks can generate a big impact in the accuracy of the model [23,24]. This work considers the use of some optimizers for training the ANNs. Following, there is a brief description of each of them. Let $g_{t}$ be the gradient at time step *t*, $\theta_{t}$ be the parameter vector, η the learning rate, and ϵ a small constant to prevent division by zero.

- RMSprop (Root Mean Square Propagation) is an adaptive learning rate optimizer that adjusts the step size for each parameter, making it effective for non-stationary objectives and widely used in recurrent neural networks. It maintains an exponentially decaying average of squared gradients ($v_{t}$) to adapt the learning rate [25].
  (3) $$v_{t}=\beta_{2}v_{t-1}+\left(1-\beta_{2}\right)g_{t}^{2}$$
  (4) $$\theta_{t+1}=\theta_{t}-\frac{\eta }{\sqrt{v_{t}}+\epsilon }g_{t}$$
- Adam (Adaptive Moment Estimation): This algorithm combines the benefits of RMSprop with momentum by maintaining exponentially decaying averages of past gradients and squared gradients, offering robust performance across various deep learning tasks. It tracks both the first moment ($m_{t}$, similar to momentum) and the second moment ($v_{t}$, similar to RMSprop). Crucially, it includes a bias correction mechanism (${\hat{m}}_{t},{\hat{v}}_{t}$) to account for the fact that $m_{t}$ and $v_{t}$ are initialized to zero, leading to biased estimates in early steps [26].
  (5) $$m_{t}=\beta_{1}m_{t-1}+\left(1-\beta_{1}\right)g_{t}$$
  (6) $$v_{t}=\beta_{2}v_{t-1}+\left(1-\beta_{2}\right)g_{t}^{2}$$
  (7) $${\hat{m}}_{t}=\frac{m_{t}}{1-\beta_{1}^{t}},\;{\hat{v}}_{t}=\frac{v_{t}}{1-\beta_{2}^{t}}$$
  (8) $$\theta_{t+1}=\theta_{t}-\frac{\eta }{\sqrt{{\hat{v}}_{t}}+\epsilon }{\hat{m}}_{t}$$
- Nadam (Nesterov-accelerated Adaptive Moment Estimation): This optimizer extends Adam by incorporating Nesterov momentum, which can lead to faster convergence in some settings. It achieves faster convergence by looking ahead, applying the momentum step before calculating the gradient [27]. The update rule is more complex but is often implemented by modifying the gradient used in the Adam update:
  (9) $$\theta_{t+1}=\theta_{t}-\frac{\eta }{\sqrt{{\hat{v}}_{t}}+\epsilon }\left(\beta_{1}{\hat{m}}_{t}+\frac{\left(1-\beta_{1}\right)g_{t}}{1-\beta_{1}^{t}}\right)$$
- AdamW: This algorithm modifies Adam by decoupling weight decay from the gradient update, improving regularization and generalization. AdamW fixes this by decoupling the weight decay (λ) from the adaptive gradient calculation, applying it as a standard decay term after the update [28].
  (10) $$\theta_{t+1}=\theta_{t}-\eta \left(\frac{{\hat{m}}_{t}}{\sqrt{{\hat{v}}_{t}}+\epsilon }+\lambda \theta_{t}\right)$$
- Lion (EvoLved Sign Momentum) is a recently introduced optimizer that emphasizes simplicity and efficiency by relying on sign-based parameter updates, achieving competitive results with fewer computational resources [29]. It only tracks the first moment ($m_{t}$) and uses the sign of the momentum update direction ($u_{t}$) for the step, resulting in a low-frequency, aggressive update. It typically requires a smaller learning rate and is computationally simpler.
  (11) $$m_{t}=\beta_{1}m_{t-1}+\left(1-\beta_{2}\right)g_{t}$$
  (12) $$u_{t}=\mathrm{sign}\left(\beta_{1}m_{t-1}+\left(1-\beta_{2}\right)g_{t}\right)$$
  (13) $$\theta_{t+1}=\theta_{t}-\eta u_{t}$$

## 3. Materials and Methods

**Figure 2:**
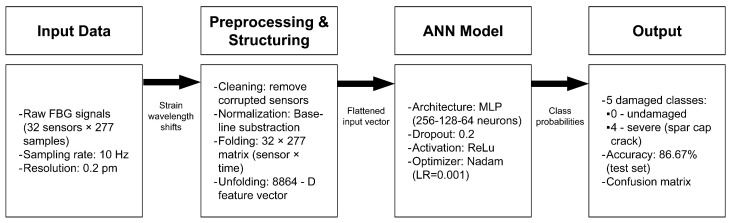
Damage Classification Pipeline [20].

The damage classification methodology is presented in Figure 2. More details about the steps and benchmarking methodology are presented in the following subsections.

### 3.1. Input Data and Data Preprocessing

The data used in this work were collected from a composite UAV wing equipped with a 32 Fiber Bragg Grating (FBG) sensors as described in Section 3. The dataset comprises 210 experiments corresponding to five structural states with progressive damage severity, each experiment produced a strain matrix of 32 × 277 samples representing the spatial and temporal distribution of the sensor network response under controlled bending loads.

The preprocessing stage aimed to transform the raw strain signals from the 32 FBG sensors into a structured and normalized input for the neural model. Each experiment generated a matrix of 32 × 277 samples, corresponding to the spatial and temporal distribution of strain across the composite wing. Corrupted or incomplete sensor signals were removed, and a baseline subtraction was applied to eliminate static offsets. The data were then reorganized using a D-type unfolding operation, which flattened each 2D matrix into a 1D feature vector of 8864 elements while preserving the spatial order of sensors and their temporal signatures [20]. This representation allowed the neural network to learn spatial patterns of deformation directly from the sensor network response.

Given the non-uniform baselines and sensor variability, the dataset presented a significant preprocessing challenge and to achieve stable convergence and comparability across samples, all features were standardized using Z-score normalization:

(14) $$x^{\prime }=\frac{x-\mu }{\sigma }$$

where μ and σ denote the mean and the standard deviation computed from the training set [30], this step was critical to mitigate amplitude disparities between sensors and ensure consistent learning dynamics using optimization.

Finally, the dataset was divided into training, validation and testing subsets, nine experiments per class were reserved for testing, while the remaining data were split 80/20 between training and validation using stratified sampling [7].

The complete damage classification pipeline is summarized in Figure 2, adapted from the authors’ previous work [20].

### 3.2. Benchmarking Methodology

The benchmarking process was designed to systematically evaluate the influence of key hyperparameters on the classification performance of the Multilayer Perceptron (MLP) model applied to FBG-based strain data; this methodological framework was divided into four progressive experimental phases, each addressing a different aspect of the model configuration and its robustness [31].

1. Phase 1—Optimizer screening: This first stage focused on identifying the most suitable optimization algorithm for this type of high-dimensional FBG dataset, while five optimizers were evaluated (Adam, Nadam, AdamW, RMSprop, and Lion) under the same network architecture (256–128–64 neurons) and loss function (categorical cross-entropy). Each optimizer was tested with three random seeds and two learning rates $\left(1\times 10^{-3}\;\mathrm{and}\;3\times 10^{-4}\right)$, and results were averaged across runs to assess stability [32].
2. Phase 2—Hyperparameter fine-tuning: based on phase 1 outcomes, Nadam and AdamW were selected for further exploration; a grid search strategy was implemented to analyze the interaction between learning rate and batch size, using four learning rates $(3\times 10^{-4}$–$1\times 10^{-3})$ and two batch configurations (64 and 128). Each combination was trained using early stopping criteria with 40 epochs patience, where the goal was to identify parameter regions that maximize the macro F1 score, ensuring both accuracy and class balance [33].
3. Phase 3—Architectural comparison: In the third phase, the effect of network depth and layer width on classification performance was studied, where six different architectures were tested, varying the number of hidden layers (from 3 to 10) and the number of neurons per layer (64–512). This analysis quantified how increasing model complexity affects generalization when the dataset exhibits sensor redundancy and high correlation between strain channels [34].
  Regularization through dropout (0.2) and L2 weight penalty $\left(1\times 10^{-4}\right)$ was maintained constant across all configurations to ensure fair comparison.
4. Phase 4—Robustness analysis: finally, the robustness performing model was assessed under controlled perturbations to emulate sensor faults and signal degradation scenarios. Three perturbation types were introduced:
  - Sensor dropout, removing a fraction of FBG channels;
  - Gaussian noise, simulating thermal or interrogation noise;
  - Linear drift, simulating calibration offset in selected sensors.
  For each condition, the model’s macro F1 score was evaluated to quantify resilience to measurement uncertainty [33].

This benchmarking structure provides a reproducible framework for analyzing how optimization algorithms, hyperparameters, and architectural complexity interact in the context of composite structure SHM using FBG sensors The four-phase methodology also highlights the trade-off between accuracy, computational efficiency, and robustness, which are essential parameters for real-time applications in aerospace monitoring systems [22].

This section describes in detail the composite UAV wing used in the experimental campaign, the materials and internal structure of the specimen, the layout and installation of the Fiber Bragg Grating (FBG) network, and the loading and boundary conditions under which the dataset was acquired. The description consolidates and extends previous works that used the same wing section and experimental bench, originally designed at TU Berlin for Boeing Research and Technology Europe under the SINTONIA project, and later employed by Sierra-Pérez and collaborators for research in Structural Health Monitoring (SHM) using optical fiber sensors. The information reported here unifies the material and geometric details from those works with the specific configuration of the dataset employed in the present study.

### 3.3. Experimental Setup

The specimen corresponds to the outboard half of a UAV wing root section with a span of 1.5 m and an average chord of 0.25 m. The structure is a multimaterial sandwich configuration that combines carbon fiber-reinforced polymer (CFRP) and glass fiber-reinforced polymer (GFRP) skins, PVC foam and balsa wood as core materials and adhesive epoxy joints bonding the layers. The internal architecture includes a main spar, secondary ribs and foam sections forming a lightweight but stiff aerodynamic structure. These elements provide a realistic representation of small-scale composite UAV wings used in research and industrial applications.

The wing root was rigidly attached to a steel test frame that replicated the fuselage interface. This fixation used an aluminium profile and four bolts with mechanical clamps to ensure stable an repeatable boundary conditions during loading, the specimen also mounted with the intrados facing upward to produce a tensile strain field on the sensorized surface when loaded in bending.

The wing was instrumented with a network of 32 FBG sensors distributed along both the intrados and extrados surfaces using four optical fibers (two per surface) with eight gratings per fiber. This arrangement enabled the acquisition of both tensile and compressive strain distributions generated during bending, improving the representation of strain-field redistribution and bending modes associated with progressive structural damage. Each grating had a nominal length of 2 mm and was centered at the following Bragg wavelengths: 1529.2, 1534.6, 1539.6, 1543.9, 1549.0, 1553.7, 1558.7, and 1562.4 nm.

Data acquisition was performed with a Microp Optics Si425 swept—laser interrogator operating at a sampling frequency of 10 Hz and a wavelength resolution better than 0.2 pm. In the reference experiments conducted by Sierra-Pérez, a Luna OBR 4400 was additionally employed for distributed measurements, however, only the discrete FBG network data were used in the dataset analyzed in this work. The raw signals were later trimmed and organized as matrices of 32 × 277 points per experiment being consistent with the sensor time structure required for machine learning applications.

**Figure 3:**
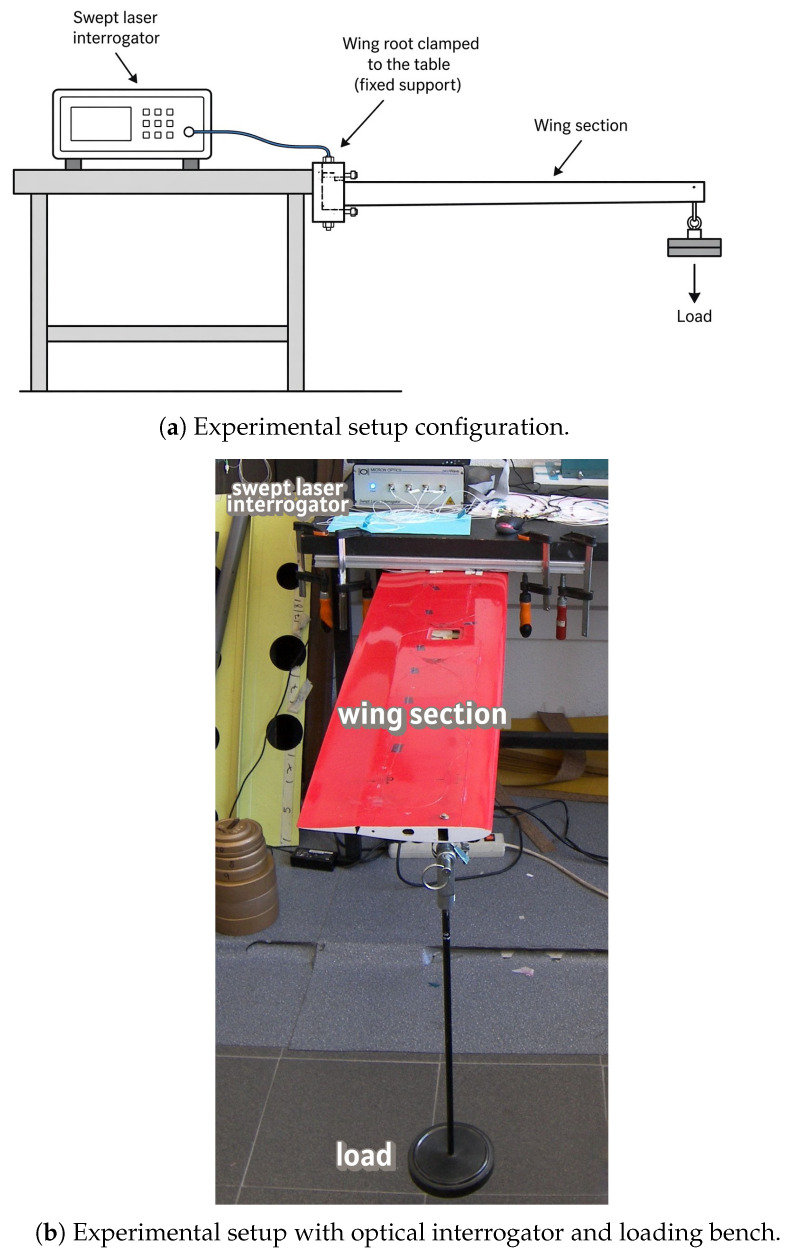
Composite UAV wing specimen and experimental setup. (**a**) Internal composition and structural configuration of the wing; (**b**) experimental bench with mounted specimen and optical interrogation system.

In total, 210 experiments were retained for analysis that correspond to five distinct structural states used in the present benchmarking study. Further details on the structure, data processing and normalization are provided in Section 3.4 and in ref. [20]. The experimental setup is illustrated in Figure 3. Panel (a) shows the experimental setup and (b) depicts the test bench and optical interrogator setup used during data acquisition; this configuration reproduces realistic boundary conditions and load transfer mechanisms representative of UAV composite wings and serves as the foundation for the sensorized structure described in Section 3.2.

### 3.4. Loading Conditions and Damage Scenarios

The experimental campaign reproduced bending conditions representative of in-flight loads. The wing section was mounted with its intrados facing upward so that the instrumented surface experienced tensile strain, while the extrados was subjected to compression. Static loads were applied at the tip of the wing using calibrated weights ranging from 3.25 to 7.25 kg, producing bending moments representative of operational conditions. Each load case was maintained for sufficient time to ensure steady state strain distribution before acquisition.

The selected load range was defined to reproduce representative bending conditions for the UAV wing while generating measurable strain redistribution throughout the FBG sensor network without inducing catastrophic structural failure. Multiple acquisitions were performed for each structural state and loading condition, contributing to the total set of 210 experiments analyzed in this study. Although the applied loading cases do not directly reproduce impact events, the induced strain redistribution patterns are representative of stiffness changes associated with barely visible impact damage (BVID), allowing the proposed SHM framework to identify damage-sensitive structural responses under controlled bending conditions.

A series of controlled damage scenarios were introduced sequentially to simulate progressive structural degradation. These included surface cuts of different lengths and orientations, as well as spar cap interruptions that locally reduced stiffness. The five structural states correspond to

1. Undamaged condition;
2. Longitudinal skin cut (3 cm);
3. Transverse cut (2 cm);
4. Transverse cut (2 cm) with a 6.5 mm spar cap cut;
5. Transverse cut (4 cm) with a 12 mm spar cap cut.

Each configuration was verified for repeatability and absence of delamination outside the intended zone.

This configuration ensured a consistent redistribution of strain throughout the sensor network, which formed the dataset used in this benchmarking study.

### 3.5. Optimizer Screening

The first phase aimed to identify the most effective optimization algorithm for classifying structural damage from FBG-based strain data. Five optimizers (RMSprop, Adam, Nadam, AdamW, and Lion) were evaluated under identical network architectures and training conditions. Each optimizer was trained using three random seeds to assess consistency and mitigate stochastic variability. The baseline architecture employed three hidden layers (256–128–64 neurons) with ReLU activations and categorical cross-entropy loss. This stage provided an initial performance reference and identified which optimizers would be further examined in the following phases.

### 3.6. Fine-Tuning

The second phase investigated the sensitivity of model performance to key hyperparameters: learning rate (LR) and batch size. Based on Phase 1, the two best-performing optimizers (AdamW and Nadam) were selected for a grid search over four learning rates $(3\times 10^{-4}$–$1\times 10^{-3})$ and two batch configurations (64 and 128). Each configuration was trained with early stopping (patience = 40 epochs) to prevent overfitting, and validation metrics were averaged across runs. The objective of this phase was to determine the learning dynamics that yield stable convergence and balanced performance across classes.

### 3.7. Architectural Comparison

The third phase assessed how network topology influences model performance. Six Multilayer Perceptron (MLP) configurations (A1–A6) were tested, ranging from shallow (three layers) to deeper architectures (up to ten layers) with neuron widths between 64 and 512. All models used the best hyperparameter settings identified in Phase 2, with fixed dropout (0.2) and L2 regularization $\left(1\times 10^{-4}\right)$ to ensure comparability. This stage aimed to understand the trade-off between model complexity, generalization capacity, and computational cost.

### 3.8. Robustness Evaluation

In the final phase, robustness was analyzed by introducing controlled perturbations to simulate sensor degradation. Three conditions were tested:

- Random sensor dropout;
- Additive Gaussian noise to mimic interrogator instability;
- Linear wavelength drift representing calibration shifts.

For the wavelength drift experiments, gradual linear perturbations of 0.5% and 1% were introduced across the sensor signals to emulate moderate calibration variations and long-term measurement instability. These perturbation levels were selected as representative controlled-drift conditions that approximate thermal fluctuations and aging-related effects commonly encountered in practical FBG-based SHM systems.

Each perturbation type was applied at incremental intensities, and the macro F1 score was monitored to evaluate how resilient the model remained under degraded measurement conditions.

## 4. Results

### 4.1. Optimizer Performance (Phase 1)

**Figure 4:**
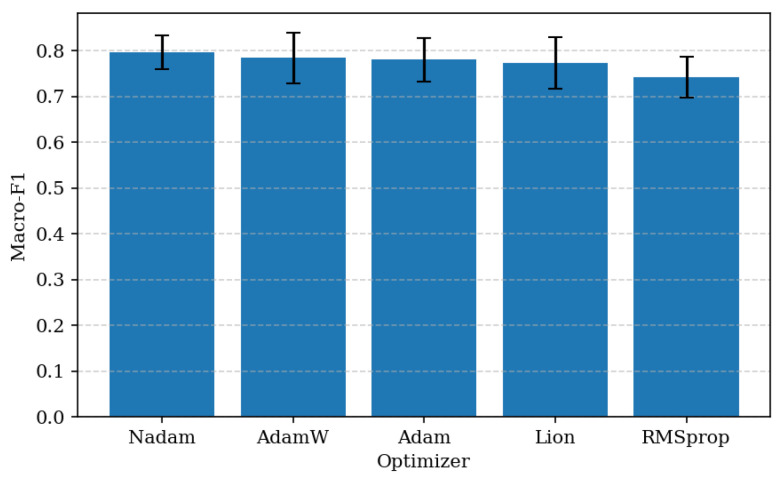
Performance comparison of five optimizers during Phase 1. Bars indicate the average Macro-F1 score across three independent runs, with error bars representing the standard deviation. AdamW and Nadam achieved the most stable and accurate results, outperforming Adam, RMSprop, and Lion.

Figure 4 summarizes the comparative performance of the five optimizers. All methods achieved macro-F1 scores between 0.80 and 0.83, confirming the stability of the baseline architecture. Among them, AdamW and Nadam delivered the highest and most consistent results (≈0.82 ± 0.02), outperforming Adam and RMSprop. This improvement is attributed to the decoupled weight decay in AdamW, enhancing generalization, and to the Nesterov momentum in Nadam, promoting smoother convergence [32,33]. The Lion optimizer, while computationally efficient, exhibited higher variability (≈0.72, $F_{1}$), likely due to its sign-based update rule being less stable for correlated sensor data. Based on these findings, AdamW and Nadam were selected for subsequent phases.

### 4.2. Hyperparameter Effects (Phase 2)

**Figure 5:**
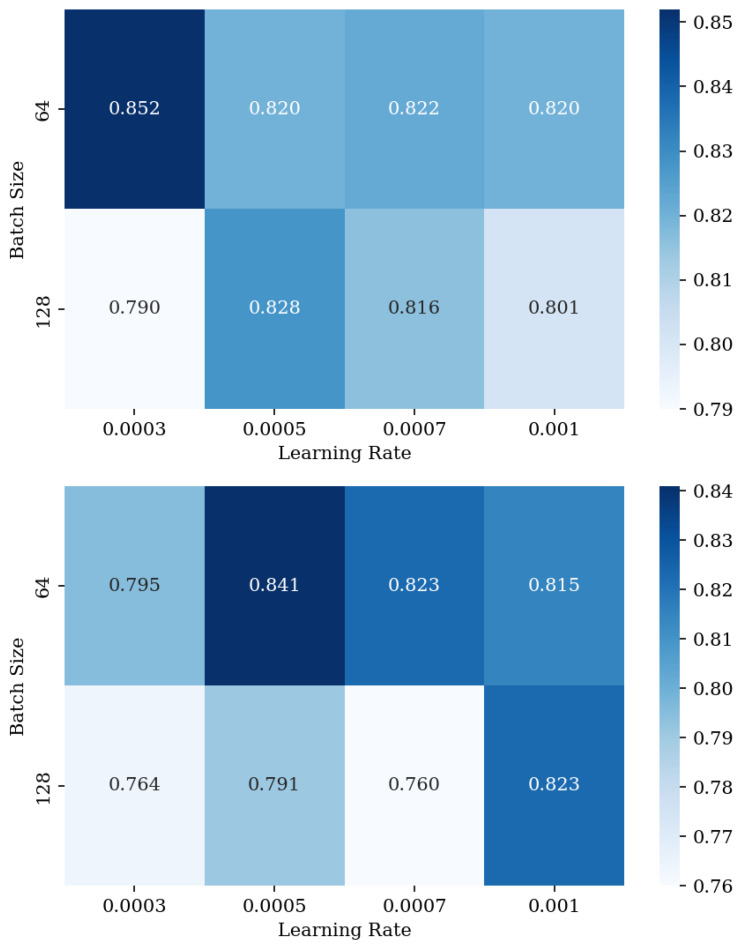
Phase 2 fine-tuning: Macro-F1 as a function of learning rate (LR) and batch size for (**top**) AdamW and (**bottom**) Nadam. AdamW achieves the best overall score (0.852) at $\mathrm{LR}=3\times 10^{-4}$, batch=64, while Nadam peaks at 0.841 with $\mathrm{LR}=5\times 10^{-4}$ and batch=64. Lower learning rates with moderate batch sizes yield the most stable performance.

The grid search analysis (Figure 5) showed that both optimizers achieved their best results with moderate batch sizes (64) and low learning rates between $3\times 10^{-4}$ and $5\times 10^{-4}$. AdamW reached a peak Macro-F1 of 0.852 at $\left(3\times 10^{-4},64\right)$, while Nadam peaked at 0.841 with $\left(5\times 10^{-4},64\right)$. These configurations provided stable convergence and balanced performance across classes, confirming that smaller learning rates help avoid oscillations during training and improve generalization on experimental datasets with correlated FBG channels.

### 4.3. Architectural Behavior (Phase 3)

The evaluated MLP topologies were designed to progressively increase architectural complexity in terms of depth and neuron density. The following configurations were considered during phase 3:

- **A1:** 256–128–64;
- **A2:** 320–160–80;
- **A3:** 256–192–128–64;
- **A4:** 320–224–160–96;
- **A5:** 256–192–144–96–64;
- **A6:** 512–384–256–128–64–32.

Architectures A1 and A2 correspond to compact shallow MLPs with progressive dimensional reduction, while A3–A6 introduce increased depth and representational capacity to evaluate the effect of the model complexity on generalization performance and robustness in FBG-based SHM classification.

**Figure 6:**
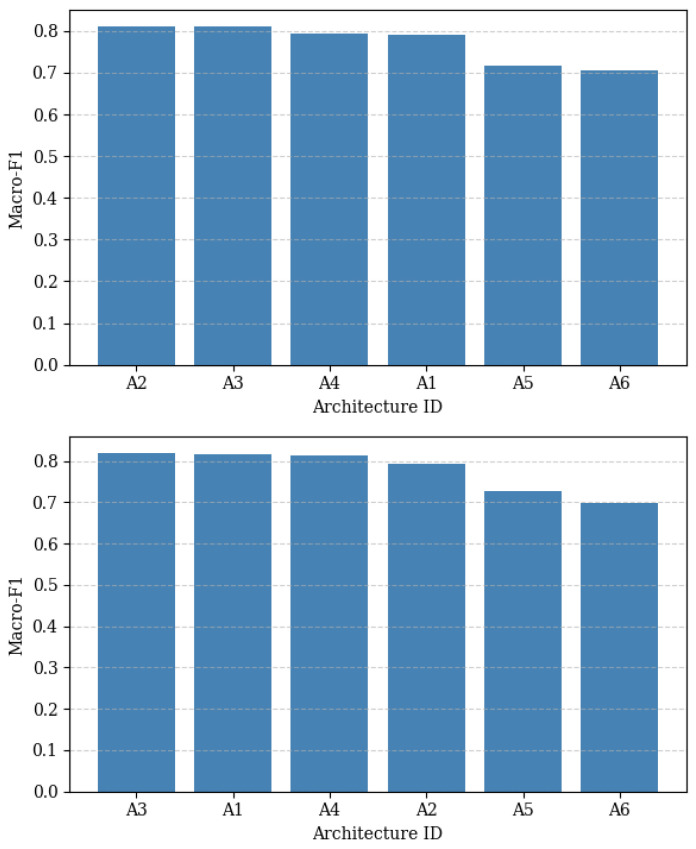
Phase 3: Architectural comparison using AdamW (**top**) and Nadam (**bottom**) optimizers. Each bar represents the mean Macro-F1 score across three runs for different Multilayer Perceptron (MLP) topologies (A1–A6). The highest performance was achieved by A2 (256–128–64 neurons) for AdamW and A3 (256–256–64 neurons) for Nadam, indicating that moderate depth yields optimal generalization for FBG-based damage classification.

The architectural comparison (Figure 6) revealed that compact MLPs with smooth dimensional reduction (A2 and A3) outperform both shallower and deeper alternatives. Specifically, AdamW achieved its best score (0.81) with architecture A2 (256–128–64), while Nadam peaked at (0.82) with A3 (256–256–64). Increasing network depth beyond four layers caused a consistent drop in performance due to overparameterization and redundancy in the strain features. These results emphasize that, for FBG-based SHM data, moderate-depth architectures capture the essential spatial strain correlations without overfitting to local sensor noise.

### 4.4. Model Robustness (Phase 4)

**Figure 7:**
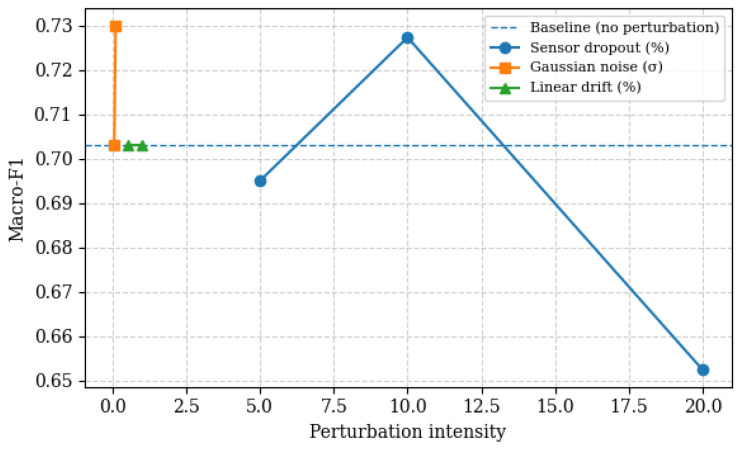
Phase 4: Robustness analysis of the best-performing model (AdamW, A2 configuration). Macro-F1 scores under increasing perturbation intensities for sensor dropout, Gaussian noise, and linear drift. The model remains stable up to 10% sensor loss and small signal distortions, demonstrating robustness suitable for real-world SHM conditions.

The robustness experiments (Figure 7) demonstrated that the best-performing configuration (AdamW, A2) maintained stable accuracy under mild signal degradation. Macro-F1 remained around 0.74 with up to 10% sensor dropout and decreased gradually to 0.65 with 20% loss. Gaussian noise (σ≤0.01 nm) and linear drifts (≤1%) produced negligible accuracy drops, indicating that the model learned global strain-field representations rather than relying on individual sensors.

This robustness is critical for real-world SHM applications where environmental and instrumentation fluctuations are inevitable. Although direct quantitative comparisons with previously published robustness studies are difficult due to differences in datasets, perturbation protocols, and sensor configurations, the observed gradual degradation under sensor dropout suggests stable distributed-strain-field learning behavior. The results indicate that the proposed MLP framework does not rely on isolated sensing channels, maintaining competitive robustness under moderate signal degradation conditions commonly encountered in practical SHM environments.

## 5. Discussion and Concluding Remarks

This study presented a systematic benchmarking framework for evaluating Multilayer Perceptron (MLP) configurations in the context of FBG-based structural damage classification in composite UAV wings. Rather than focusing solely on accuracy improvements, the analysis examined the interaction between optimization strategies, architectural complexity, and robustness under realistic measurement degradation scenarios.

The results indicate that performance saturation occurs with moderate-depth architectures, particularly configurations with progressive dimensional reduction (e.g., 256–128–64). Increasing architectural depth beyond four hidden layers did not produce consistent performance gains and, in several cases, reduced generalization capability. This behavior suggests that the strain redistribution patterns induced by progressive structural damage are sufficiently represented within compact fully connected structures, especially when the dataset exhibits high inter-sensor correlation and limited sample size.

While convolutional and recurrent architectures have demonstrated strong performance in large-scale SHM applications [12,16,18], such models are typically advantageous when spatial locality or long temporal dependencies play a dominant role. In the present experimental dataset, strain measurements correspond to quasi-static loading conditions with strong spatial correlation among sensors and limited temporal dynamics. Under these conditions, deeper architectures such as CNNs or LSTMs may introduce additional parameters without proportional gains in discriminative power. The observed stability of compact MLPs indicates that representational sufficiency can be achieved without hierarchical feature extraction mechanisms, thereby reducing computational cost and facilitating potential embedded deployment.

From the computational perspective, the recorded training times during Phase 3 also reflected the impact of architectural complexity. Compact architectures such as A1 and A2 required substantially lower computational cost while maintaining competitive Macro-F1 performance. In contrast, deeper configurations, such as A6, increased training time considerably without providing proportional gains in classification accuracy. Across all evaluated architectures, training times ranged from approximately 20 s to 120 s, depending on topology depth, optimizer configuration, and regularization settings. These observations reinforce the suitability of moderate-depth MLPs for lightweight SHM implementations and potential embedded deployment scenarios.

The robustness analysis further strengthens this observation. Performance degradation under sensor dropout occurred gradually rather than abruptly, indicating that the learned representation captures distributed strain-field patterns rather than relying on isolated sensor channels. Similarly, limited sensitivity to Gaussian noise and wavelength drift suggests that normalization and architectural regularization enable stable learning under moderate signal perturbations. This characteristic is particularly relevant for operational SHM systems, where partial sensor malfunction, calibration drift, or interrogation instability are unavoidable.

From a methodological perspective, the proposed four-phase benchmarking protocol contributes a structured and reproducible framework for analyzing neural network behavior in experimental SHM datasets. Instead of prioritizing architectural complexity, the findings emphasize the importance of balancing representational capacity, dataset size, sensor redundancy, and regularization. In experimental FBG-based SHM scenarios with moderate data volume, compact adaptive-optimizer-driven MLPs provide an efficient trade-off between accuracy, stability, and computational demand.

Nevertheless, several limitations should be acknowledged. The dataset comprises 210 experiments under quasi-static bending loads, which constrains the diversity of dynamic behavior and temporal variability. Additionally, although robustness was evaluated under controlled perturbations, real operational environments may introduce more complex multi-factor degradation mechanisms. Future research should extend this benchmarking framework to larger datasets, dynamic loading scenarios, and hybrid sensor networks, as well as investigating integration with digital twin environments for adaptive model updating.

In conclusion, the present work demonstrates that lightweight MLP architectures trained with adaptive optimizers can effectively classify structural damage states in composite UAV wings instrumented with FBG sensors. The results challenge the assumption that deeper or hybrid neural architectures are inherently superior for all SHM applications and instead highlight the value of systematic benchmarking and architectural sufficiency analysis. These findings provide practical guidance for the design of computationally efficient, robust, and deployable SHM models in aerospace systems.

## Data Availability

Dataset available on request from the authors.

## Abbreviations

The following abbreviations are used in this manuscript:

ANN	Artificial Neural Network
ANNs	Artificial Neural Networks
A2, A3	Neural network architectures tested (e.g., 256–128–64; 320–160–80)
BVID	Barely Visible Impact Damage
BOA	Butterfly Optimization Algorithm
CNNs	Convolutional Neural Networks
CFRP	Carbon Fiber-Reinforced Polymer
FBG	Fiber Bragg Grating
F1	Macro F1 Score (harmonic mean of precision and recall)
GFRP	Glass Fiber-Reinforced Polymer
GHz	Gigahertz
kg	Kilogram
L2	L2 Regularization (weight decay)
Lion	Sign-based Adaptive Optimizer
LR	Learning Rate
LSTMs	Long Short-Term Memory Networks
ML	Machine Learning
MLP	Multilayer Perceptron
Nadam	Nesterov-accelerated Adaptive Moment Estimation Optimizer
NDT	Nondestructive Testing
nm	Nanometer
OBR	Optical Backscatter Reflectometer
PSO	Particle Swarm Optimization
PVC	Polyvinyl Chloride (foam core)
ReLU	Rectified Linear Unit (activation function)
RMSprop	Root Mean Square Propagation Optimizer
SHM	Structural Health Monitoring
UAV	Unmanned Aerial Vehicle
WDM	Wavelength Division Multiplexing
AdamW	Adaptive Moment Estimation with Weight Decay Optimizer
Acc	Accuracy
BalAcc	Balanced Accuracy
